# *APOE*‐ε4×Sex×SNP Interactions in Cognition: A Multi‐Cohort GWAS Meta‐Analysis

**DOI:** 10.1002/alz70855_104456

**Published:** 2025-12-24

**Authors:** Alex G. Contreras, Skylar Walters, Jaclyn M. Eissman, Derek B. Archer, Alexandra N. Regelson, Alaina Durant, Michelle J. Clifton, Shubhabrata Mukherjee, Michael L. Lee, Seo‐Eun Choi, Emily H. Trittschuh, Lisa L. Barnes, Amanda Kuzma, Julie A Schneider, Walter W. Kukull, Sterling C Johnson, Corinne D. Engelman, Margaret Pericak‐Vance, Badri N Vardarajan, Eden R. Martin, Brian W Kunkle, Jonathan L Haines, Li‐San Wang, Richard Mayeux, Gerald D. Schellenberg, Michael L Cuccaro, David A. A. Bennett, Paul K Crane, Timothy J. Hohman, Logan Dumitrescu

**Affiliations:** ^1^ Vanderbilt Memory & Alzheimer's Center, Nashville, TN, USA; ^2^ Vanderbilt Genetics Institute, Nashville, TN, USA; ^3^ Vanderbilt Memory & Alzheimer's Center, Vanderbilt University Medical Center, Nashville, TN, USA; ^4^ Columbia University Irving Medical Center, New York, NY, USA; ^5^ Vanderbilt University Medical Center, Nashville, TN, USA; ^6^ Vanderbilt Memory and Alzheimer's Center, Vanderbilt University Medical Center, Nashville, TN, USA; ^7^ University of Washington Alzheimer's Disease Research Center, University of Washington School of Medicine, Seattle, WA, USA; ^8^ University of Washington, Seattle, WA, USA; ^9^ University of Washington, School of Medicine, Seattle, WA, USA; ^10^ Rush Alzheimer's Disease Center, Rush University Medical Center, Chicago, IL, USA; ^11^ Penn Neurodegeneration Genomics Center, Perelman School of Medicine, University of Pennsylvania, Philadelphia, PA, USA; ^12^ Rush Alzheimer's Disease Center, Department of Neurological Sciences, Rush University Medical Center, Chicago, IL, USA; ^13^ University of Wisconsin‐Madison School of Medicine and Public Health, Madison, WI, USA; ^14^ University of Miami Miller School of Medicine, Miami, FL, USA; ^15^ Taub Institute for Research on Alzheimer's Disease and the Aging Brain, Columbia University, New York, NY, USA; ^16^ University of Miami, Miller School of Medicine, John P. Hussman Institute for Human Genomics, Miami, FL, USA; ^17^ Case Western Reserve University School of Medicine, Cleveland, OH, USA; ^18^ University of Pennsylvania Perelman School of Medicine, Philadelphia, PA, USA; ^19^ Columbia University Medical Center, New York, NY, USA; ^20^ University of Washington Alzheimer's Disease Research Center, Seattle, WA, USA; ^21^ Vanderbilt Genetics Institute, Vanderbilt University Medical Center, Nashville, TN, USA; ^22^ Vanderbilt Memory and Alzheimer's Center, Vanderbilt University School of Medicine, Nashville, TN, USA

## Abstract

**Background:**

Two‐thirds of Alzheimer's disease (AD) patients are women, and sex‐specific *APOE*‐related cognitive impairment is well‐documented. However, the interplay among *APOE* genotype, sex, and common genetic variants (SNPs) remains poorly understood, particularly for specific cognitive domains. We performed a GWAS meta‐analysis to investigate the SNP×*APOE*‐ε4×sex interaction on memory, leveraging harmonized data from multiple cohorts. Future analyses will address executive function and language.

**Method:**

We aggregated data from six cohorts (ACT, MAP, NACC, NIAADFBS, ROS, and WRAP), with plans to incorporate additional cohorts in the final analysis. All participants had genome‐wide SNP data and harmonized memory scores derived via confirmatory factor analysis. Our current sample includes 33,440 European‐ancestry individuals, with final analyses expanding to African and other ancestries. *APOE* status was determined by genotyping rs7412 and rs429358. Our meta‐analysis focused on the three‐way interaction (SNP×*APOE*‐ε4×sex) for memory. In addition, we ran four main‐effects GWAS meta‐analyses stratified by sex and *APOE*‐ε4 status (female carriers, female non‐carriers, male carriers, male non‐carriers), adjusting for age and principal components. Two‐way interaction analyses (SNP×*APOE*‐ε4, SNP×sex) are planned for future analyses.

**Result:**

Preliminary analyses identified 112 SNPs with suggestive associations (*p* <5×10^‐6^) for the three‐way interaction on memory. Notably, one locus is intronic to *HGSNAT*, a gene involved in lysosomal function and implicated in cognition. Because *APOE*‐ε4 is also linked to lysosomal dysfunction and neuroinflammation, these findings may suggest a novel connection between *HGSNAT* and *APOE*‐ε4 in cognitive decline. Stratified GWAS revealed four genome‐wide significant main‐effect signals in male *APOE*‐ε4 carriers for memory. The most significant signal was rs76307224 (β_male‐carriers_=‐2.2, P_male‐carriers_=2.18×10^‐10^), which approached nominal significance in male non‐carriers (β_male‐noncarriers_=‐0.01, P_male‐noncarriers_=0.07) but was insignificant in females regardless of *APOE* genotype (*p*'s >0.5).

**Conclusion:**

By modeling these interactions, our study aims to uncover novel biological pathways underlying cognitive decline. Our preliminary findings highlight the potential for identifying SNP associations with cognitive performance that are modulated by sex and *APOE* status. Expanding the dataset with additional cohorts, diverse ancestries, and other cognitive domains will enhance statistical power and generalizability. Future work will assess whether these interaction effects persist longitudinally and vary by ancestry, offering deeper insights into AD risk.

